# Structural anterior segment OCT biomarkers do not predict secondary intervention after Preserflo MicroShunt implantation

**DOI:** 10.3389/fmed.2026.1763430

**Published:** 2026-04-23

**Authors:** Martin Kallab, Sarah Hinterberger, Sophie Schneider, Olivia Murauer, Anna-Sophie Reisinger, Susanne Strohmaier, Alex S. Huang, Matthias Bolz, Clemens A. Strohmaier

**Affiliations:** 1 Department of Ophthalmology and Optometry, Kepler University Hospital, Johannes Kepler University, Linz, Austria; 2 Department of Epidemiology, Center for Public Health, Medical University of Vienna, Vienna, Austria; 3 The Viterbi Family Department of Ophthalmology, Hamilton Glaucoma Center, Shiley Eye Institute, University of California, San Diego, San Diego, CA, United States

**Keywords:** anterior segment optical coherence tomography, anterior segment optical coherence tomography angiography, bleb failure, filtration surgery, open angle glaucoma, Preserflo MicroShunt

## Abstract

**Purpose:**

This study aimed to evaluate the anterior segment optical coherence tomography (AS-OCT) parameters bleb wall thickness (BWT) and total bleb height (TBH) in the early postoperative phase after Preserflo MicroShunt (PM) implantation for their correlation with (a) secondary interventions and (b) AS-OCT angiography (AS-OCTA)-derived bleb vessel density (BVD).

**Materials and methods:**

In a prospective longitudinal study of 23 open-angle glaucoma patients, AS-OCT measurements of BWT and TBH and AS-OCTA measurements of BVD were obtained at 1, 2, and 4 weeks (1 W, 2 W, and 4 W), as well as at 2, 3, 6, 9, and 12 months postoperatively. Secondary interventions (needling or open revision) were recorded. The correlations of BWT and TBH with (a) secondary interventions and (b) BVD were assessed.

**Results:**

A total of 10 of 23 patients needed secondary interventions between 4 and 48 weeks after PM implantation. At 1, 2, and 4 weeks after surgery, neither BWT nor TBH was significantly associated with future secondary interventions (BWT/TBH *p*-values: 1 W: 0.217/0.878, 2 W: 0.670/0.528, and 4 W: 0.171/0.430). Similarly, no correlations between BWT or TBH and BVD were found.

**Conclusion:**

Structural AS-OCT-based bleb thickness parameters (BWT and TBH) in the early postoperative phase were not significantly associated with future secondary interventions after PM implantation and did not correlate with AS-OCTA-derived BVD. BVD, which was shown to be a viable biomarker for future secondary interventions in a previous analysis of the same cohort, could be a better predictor of secondary interventions after PM implantation than BWT/TBH. Larger studies are necessary to confirm these hypothesis-generating findings.

## Introduction

Lowering intraocular pressure (IOP) is still the mainstay of glaucoma treatment. Surgical options to lower IOP have seen numerous advancements toward a more standardized and potentially safer approach to filtration surgery. Still, a considerable number of patients need secondary interventions (1), and therefore, an intensified follow-up regime is required for optimal patient care (2).

Filtration surgeries aim at generating an aqueous humor bypass from the anterior chamber to the subconjunctival/subtenon space, either by creating a surgical scleral flap (trabeculectomy [TE]) or through implant-assisted procedures such as Xen gel (XG) implantation (3, 4) or, more recently, Preserflo MicroShunt (PM) implantation (5, 6). While TE has consistently shown greater IOP-lowering efficacy than PM implantation, PM implantation is still associated with a marked IOP reduction that is close to TE and has a favorable perioperative and postoperative risk profile (7–9). As a result, PM implantation is increasingly being used as a first-line glaucoma surgery as opposed to traditional TE (7–9).

Filtration surgeries, in general, depend on proper filtration bleb (FB) function, and, therefore, FB evaluation, either by clinical observation or by anterior segment imaging, plays a central role in postoperative patient management. The value of clinical bleb grading scales, e.g., the Moorfields Bleb Grading System (MBGS) (10) and the Indiana Bleb Appearance Grading Scale (IBAGS) (11), is, however, impaired by partly subjective grading algorithms (12, 13). They also lack a detailed evaluation of internal bleb structure and dimensions. To overcome these limitations, anterior segment optical coherence tomography (AS-OCT) has been used to develop objective biomarkers for bleb function (14). Currently, bleb wall thickness (BWT) and total bleb height (TBH) are among the most frequently studied quantitative AS-OCT-based structural bleb parameters and have been repeatedly shown to correlate well with bleb function after TE (15–22). The results when examining the same endpoints after PM implantation have been equivocal (23–25).

Moreover, while existing cross-sectional data on the correlation between concomitantly recorded AS-OCT parameters and IOP undoubtedly help to extend our knowledge of pathophysiological processes associated with bleb failure comprising vascularization, fibrosis, and bleb encapsulation, their utility in clinical routine care may, however, be limited. From a clinical perspective, biomarkers informing the clinician early about future bleb failure risk are needed to allow a personalized approach to glaucoma patient care. For the above-mentioned quantitative AS-OCT-based bleb parameters, information on their predictive value is, however, scarce after TE (15, 16) and is not available after PM implantation. As we recently identified AS-OCT angiography (AS-OCTA)-measured bleb vessel density (BVD) at 2 and 4 weeks after surgery as a predictor for secondary intervention up to 1 year after PM primary surgery (26), we set out to investigate this relationship for structural AS-OCT bleb parameters in the same cohort.

Therefore, this study aimed to evaluate structural AS-OCT bleb parameters, BWT and TBH, in the early postoperative phase with respect to their predictive value for secondary intervention after PM implantation in a longitudinal study cohort and to correlate those parameters with AS-OCTA-derived bleb vascularity information.

## Methods

### Study design and patient selection

The protocol of this single-center study, which was conducted at the Department of Ophthalmology and Optometry, Kepler University Hospital, Linz, Austria, was approved by the Ethics Committee of the Johannes Kepler University (EC-No.: B-142-17). Written informed consent was obtained from every patient before study inclusion, and the tenets of the Declaration of Helsinki were followed during all study-related procedures. Patient recruitment took place from 6 August 2020 to 10 November 2022.

The inclusion criteria were glaucoma type (primary open-angle glaucoma, pseudoexfoliation glaucoma, or pigment dispersion glaucoma) and indication for PM implantation (maximal tolerated IOP-reducing medical therapy and uncontrolled IOP > 21 mmHg and/or visual field [VF] progression, as tested using the 30–2 SITA fast algorithm of Humphrey Field Analyzer II 750 [Carl Zeiss Meditec Inc., Dublin, CA, USA]), and/or progressive retinal nerve fiber layer thickness reduction, as measured using Spectralis OCT (Heidelberg Engineering, Heidelberg, Germany). The exclusion criteria were angle-closure glaucoma and previous filtration surgery.

### Preserflo MicroShunt: medical device and implantation surgery

Descriptions of in-depth surgical technique and specifications of the PM medical device (Santen Pharmaceutical, Osaka, Japan) have been published elsewhere (5, 6). Briefly, the PM glaucoma drainage device is made from poly(styrene-block-isobutylene-block-styrene) (SIBS), a highly biocompatible and bioinert material, and has the following basic dimensions: length 8.5 mm, outer diameter 350 μm, and inner diameter 70 μm.

In this study, standard mitomycin C (MMC)-augmented PM implantation was performed in the superior-temporal or superior-nasal quadrant under subtenon or general anesthesia. MMC-soaked sponges (0.02 mg/mL) were applied for 3 min after dissection of the conjunctiva before the scleral pocket and tunnel to the anterior chamber were created. Then, the PM was placed in the tunnel with its fins inside the scleral pocket. Finally, the implant was primed by balanced saline solution injection, and flow was verified by visual inspection. Conjunctival wound closure was performed using interrupted, resorbable sutures.

### Postoperative treatment and examinations

Preservative-free antibiotic (ofloxacin, 1 week) and steroid (dexamethasone, tapered over 12 weeks) drops were prescribed according to a standardized protocol. Slit-lamp examinations, IOP measurements, structural AS-OCT measurements (Casia 2 Cornea/Anterior Segment OCT, Tomey Corporation, Nagoya, Japan), and AS-OCTA measurements (PLEX Elite 9,000; Carl Zeiss Meditec, Dublin, CA, USA) were performed at 1, 2, and 4 weeks, as well as at 2, 3, 6, 9, and 12 months after surgery. Moreover, the necessity and number of IOP-lowering medications (MEDs), as well as secondary interventions (needling or open revision), were recorded. Experienced clinicians (S. SCH, A-S. R., and CA. S.) offered a secondary intervention if postoperative IOP exceeded the preoperatively defined target pressure and no bleb or a scarred bleb was clinically visible. No bleb was needled/revised based on clinical bleb appearance only. Prescription of IOP-lowering medications was not considered as an alternative first-line approach without prior needling/revision. All clinicians involved in secondary intervention decision-making were blinded to the imaging results.

Based on whether the endpoint “secondary intervention” was met, defined as the need for either needling or open revision, patients were allocated to the outcome groups “secondary intervention” or “no secondary intervention.” This approach needs to be differentiated from classification based on a predetermined IOP threshold (e.g., −20% IOP reduction needed for success).

### AS-OCT(A) scan acquisition and image analysis

**Figure 1:**
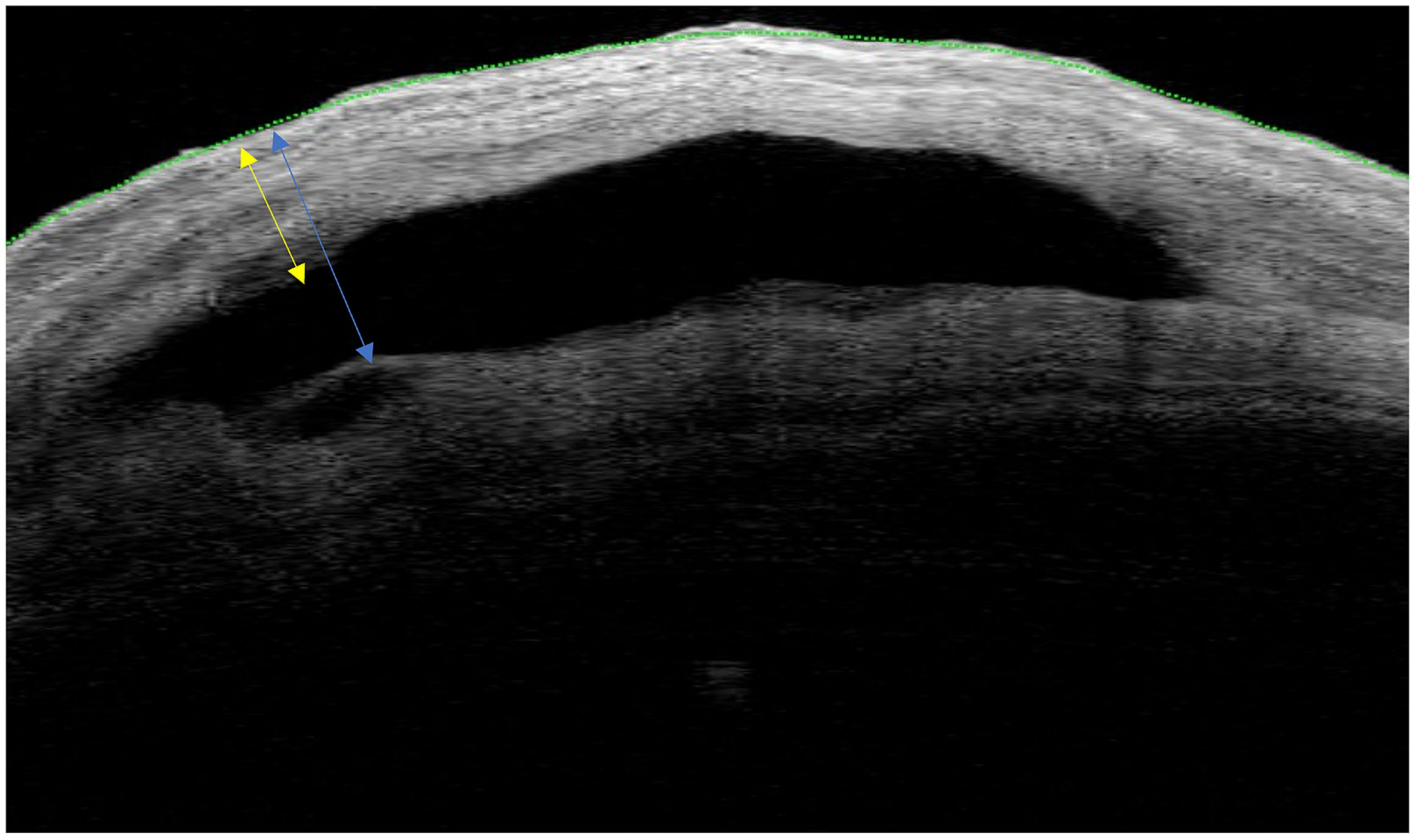
Illustration of the measurements acquired for TBH (blue arrow) and BWT (yellow arrow). Both biomarkers were acquired at the location of the distal tip of the PM implant.

All AS-OCT images were obtained using the Casia2 AS-OCT with on-board software. Operators instructed the patients to look down to expose the FB in the superior-nasal or superior-temporal quadrant. Upon visualization of the FB and PM in the real-time preview OCT image, an AS-OCT volume was acquired using the standard settings of the “Bleb” imaging mode (volume size 12×12 mm, number of B-scans: 256, number of A-scans per B-scan: 400). Image analysis was performed by one author (S. H.) trained in AS-OCT image analysis who was blinded to the clinical outcome. To measure BWT and TBH, a single B-scan of one volume scan per timepoint was selected based on the visibility of the following structures to ensure intra- and inter-individual comparability: posterior PM tip, complete bleb wall above PM, and episcleral fluid. If more than one scan fulfilled all criteria, the B-scan with the higher bleb was selected. If no episcleral fluid was visible in any scan (i.e., not present in the individual bleb), this criterion was omitted. To ensure the consistency of selected bleb regions across study visits, the most comparable B-scan at every study visit was selected based on PM configuration in the B-scan and position of the individual B-scan overlay on the near-infrared enface image, as generated by default by onboard software. Figure 1 illustrates the acquisition of bleb parameters.

**Figure 2:**
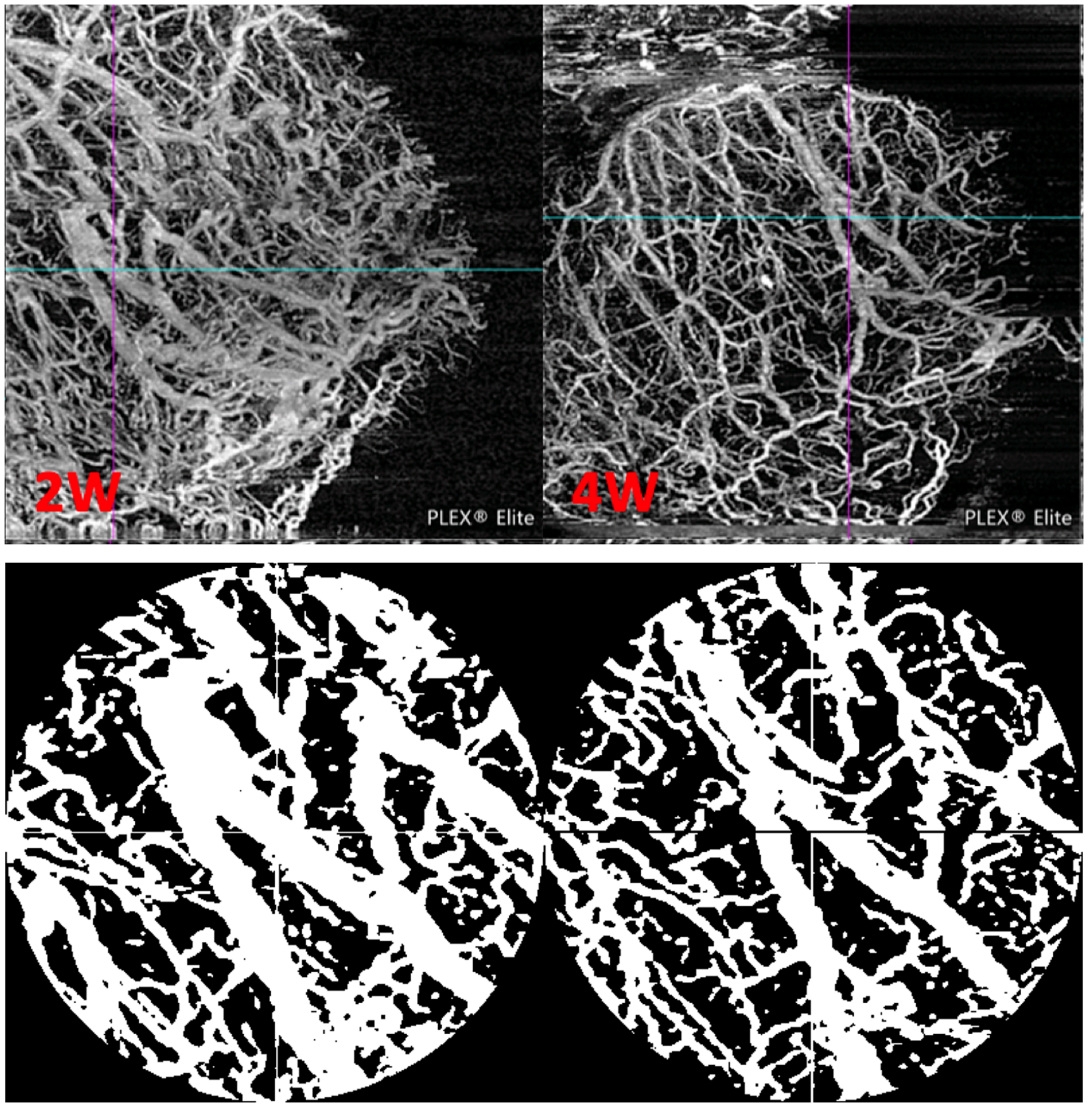
Representative images illustrating the BVD measurements. The top panels show the raw, unprocessed en-face OCTA images of a filtration bleb (2 and 4 weeks after PM surgery). The cyan/magenta crosshair marks the PM location as determined in the B scans. The bottom panels show the corresponding processed images (artifact removal, binarization, and ROI selection), as they were used for analysis. Modified from Schneider et al. (26) with permission.

All AS-OCTA images were acquired using the PLEX Elite 9,000 OCTA in combination with a 10-dpt anterior segment add-on lens. Details of our AS-OCTA image processing and BVD calculation approach were published previously (26). In short, AS-OCTA slabs with the PM scleral passage position marked in an overlay were exported. Using Fiji ImageJ (27), motion artifacts were removed before a circular region of interest (400px diameter) centered around the scleral passage of the PM was defined. The images were then binarized, and the BVD was calculated as the proportion of white pixels (Figure 2 illustrates the image analysis process).

### Statistical analysis

If not differently indicated, all values were presented as means±standard deviation (SD). Normal distribution of values was confirmed using the Shapiro–Wilk test. Logistic regression modelling in combination with the likelihood-ratio test was used to evaluate predictive values of independent variables (BWT or TBH) for the dependent dichotomic variable (secondary intervention and no secondary intervention). The Pearson bivariate correlation was used to evaluate associations of structural AS-OCT parameters (BWT and TBH) with AS-OCTA parameters (BVD). Prism 10 (GraphPad Software, Boston, MA, USA) was used for statistical testing and graph production. R (RStudio) (28) was used for post-hoc power calculation.

## Results

### Baseline characteristics

A total of 23 patients with OAG and preoperative IOP of 23.57 ± 7.75 mmHg received PM implantation and were included in this study. In ten patients, needling or open revision was performed during the 1-year follow-up period. The earliest secondary interventions were performed after the 4-week postoperative follow-up. Further baseline characteristics in the whole cohort and the two outcome groups are summarized in Table 1.

**Table 1:** Baseline characteristics.

	Whole cohort	SI	No SI
*N*	23	10	13
Age (mean ± SD) (years)	69.26 ± 10.05	68.2 ± 3.99	70.08 ± 13.09
Sex (female/male)	13/10	5/5	8/5
Ethnicity (Caucasian/Asian)	22/1	9/1	13/0
OAG type (POAG/PDG/PXG)	17/1/5	9/0/1	8/1/4
MD (dB, mean ± SD)	−9.83 ± 7.43	−6.13 ± 5.74	−12.67 ± 7.52
IOP-preOP (mmHg)	23.57 ± 7.75	24.40 ± 8.80	22.92 + 7.15
MEDs-preOP (mean ± SD)	2.96 ± 1.11	3.40 ± 0.52	2.62 ± 1.33
IOP-postOP (1 W) (mmHg)	8.30 ± 2.12	9.60 ± 1.84	7.31 ± 1.80
Timepoint of SR (week, median, and minimum–maximum)	—	10, 4–48	—

SR, secondary intervention; OAG, open-angle glaucoma; POAG, primary open-angle glaucoma; PDG, pigment dispersion glaucoma; PXG, pseudoexfoliation glaucoma; IOP, intraocular pressure measured using Goldmann applanation tonometry; MEDs, number of topical medication classes (this included topical prostaglandin analogs in all except for one patient). MD, visual field mean deviation; dB, decibel; 1 W, 1 week.

### Descriptive statistics of postoperative IOP, BWT, and TBH

**Figure 3:**
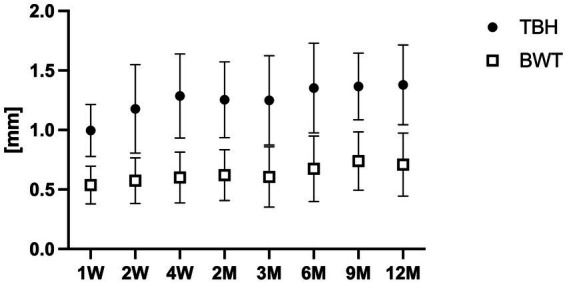
Time course of bleb wall thickness (BWT) and total bleb height (TBH). TBH: total bleb height, BWT: bleb wall thickness.

Postoperative IOP was 8.30 ± 2.12, 9.17 ± 2.33, 11.70 ± 4.39, 13.48 ± 5.83, 11.87 ± 4.49, 12.30 ± 6.65, 11.87 ± 3.11, and 13.05 ± 4.12 mmHg at 1, 2, and 4 weeks, as well as at 2, 3, 6, 9, and 12 months after surgery. Postoperative BWTs and TBHs over time are summarized in Table 2 and Figure 3.

**Table 2:** Tabulated time course of TBH and BWT values.

Postoperative timepoint	TBH [μm]	BWT [μm]
1 week	996 ± 218	538 ± 159
2 weeks	1,177 ± 373	574 ± 192
4 weeks	1,286 ± 354	601 ± 214
2 months	1,255 ± 319	621 ± 213
3 months	1,249 ± 375	607 ± 254
6 months	1,353 ± 377	674 ± 276
9 months	1,366 ± 281	739 ± 246
12 months	1,379 ± 335	709 ± 266

TBH, total bleb height; BWT, bleb wall thickness. Values are presented as means ± SD.

### Association of BWT and TBH with future secondary intervention

**Figure 4:**
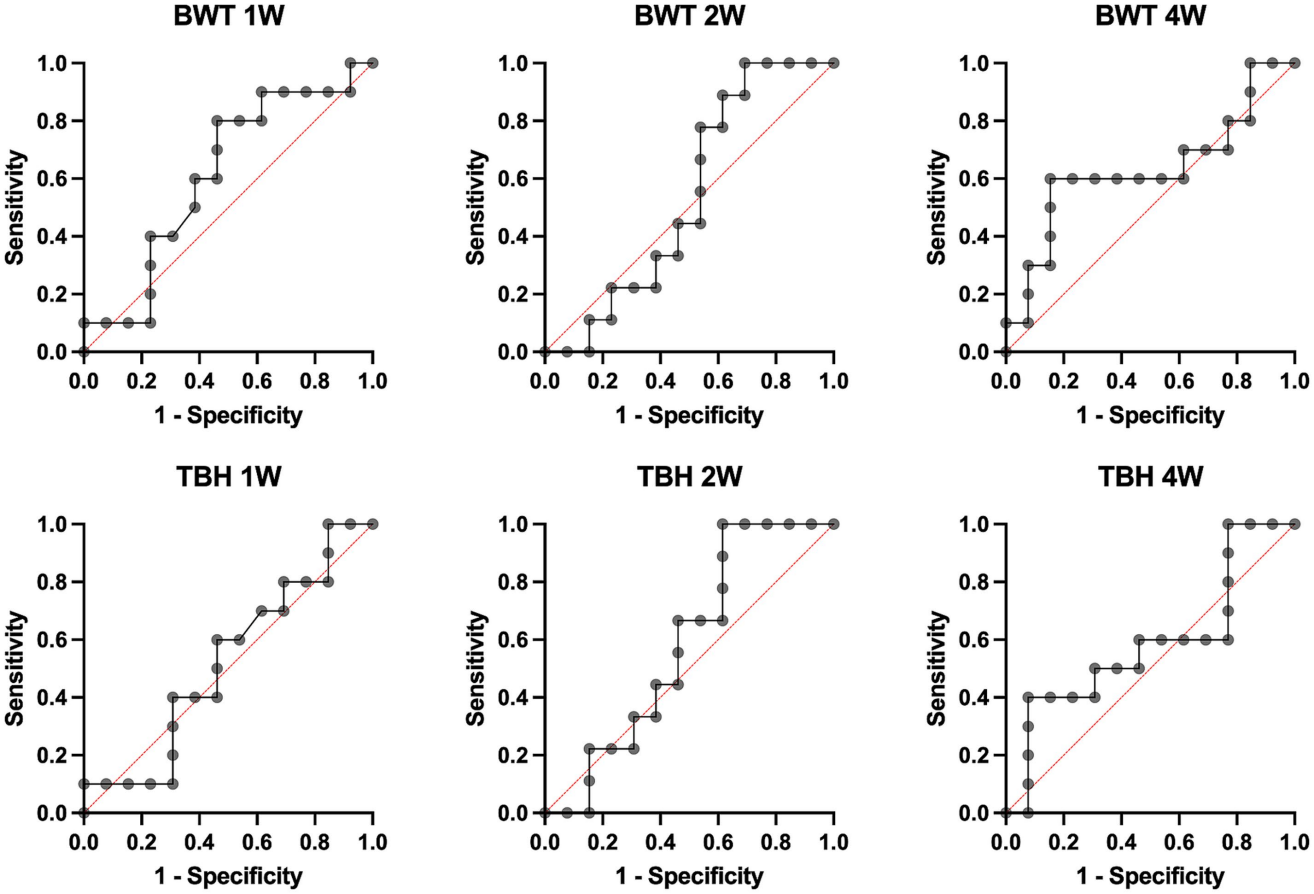
Receiver operating characteristic curves for bleb wall thickness (BWT) and total bleb height (TBH) 1, 2, and 4 weeks (1 W, 2 W, and 4 W) after primary surgery.

As the earliest secondary interventions were performed after the 4-week postoperative follow-up, BWT and TBH were evaluated at 1, 2, and 4 weeks. Logistic regression models revealed that neither BWT nor TBH was significantly associated with future secondary interventions at 1, 2, and 4 weeks after surgery. Areas under the receiver operating characteristic curves (AUROCs) were low for both parameters at all evaluated timepoints (BWT / TBH AUROCs [confidence interval (CI)]: 1 W: 0.612 [0.374–0.849] / 0.519 [0.276–0.762], 2 W: 0.539 [0.293–0.784] / 0.581 [0.339–0.823], and 4 W: 0.631 [0.384–0.878] / 0.585 [0.336–0.833]) and are presented in Figure 4. (BWT/TBH likelihood-ratio test *p*-values: 1 W: 0.2169/0.8776, 2 W: 0.6704/0.5281, and 4 W: 0.1706/0.4298).

Because we present negative results, we performed a *post-hoc* power analysis: Assuming a baseline secondary intervention incidence of 30%, the sample size (*n* = 23) in the present study would have allowed for the detection of a significant association if the minimum/maximum values for either parameter modified this risk by 20%.

### Correlation between structural (BWT and TBH) and vascular (BVD) data

**Figure 5:**
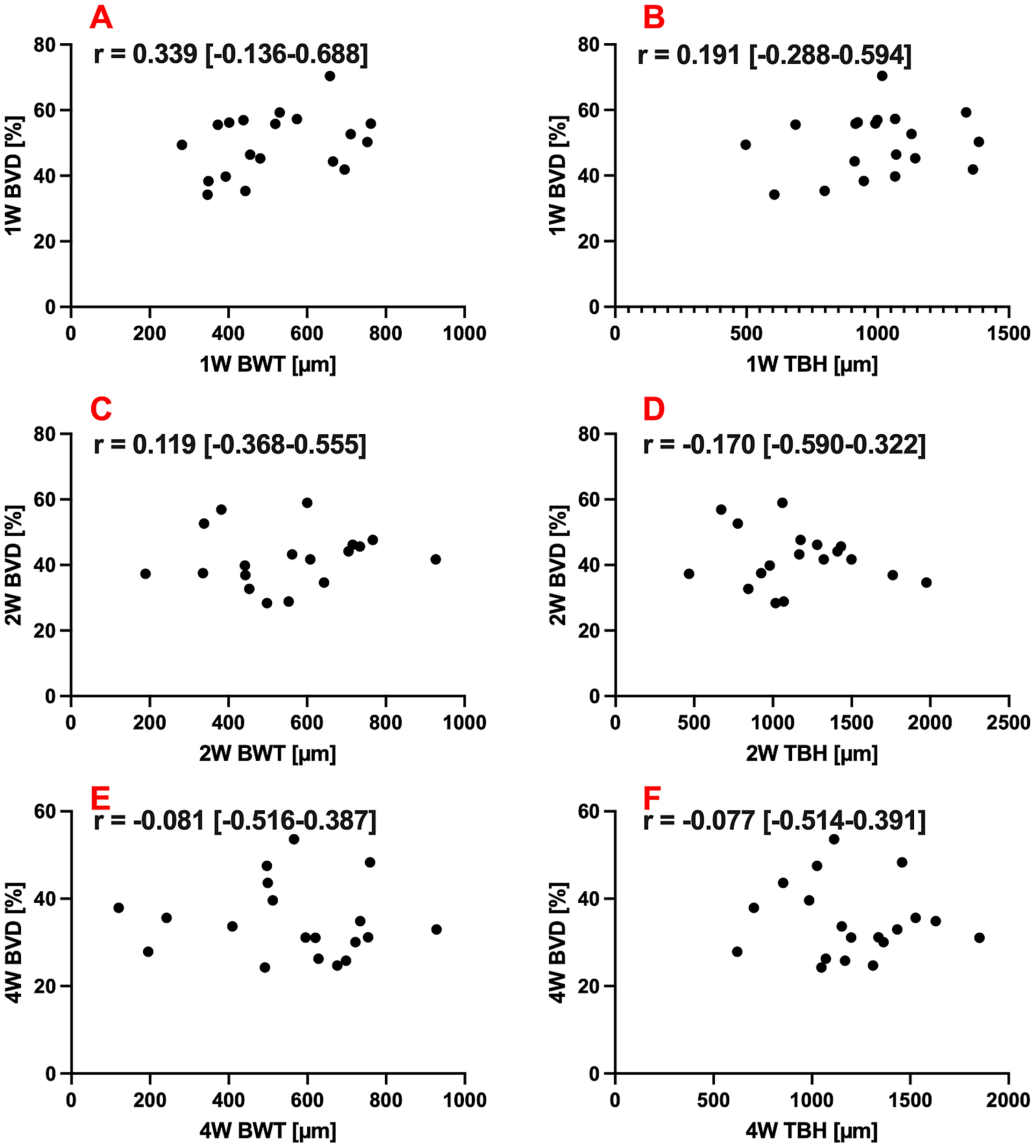
Scatter plots and correlation coefficients (*r*) with 95% confidence intervals of correlations between bleb wall thickness (BWT; **A,C,E**) or total bleb height (TBH; **B,D,F**) and bleb vessel density (BVD) 1, 2, and 4 weeks (1 W, 2 W, and 4 W) after primary surgery.

The correlations between structural parameters (BWT and TBH) and an AS-OCTA-based bleb vascularity parameter (BVD), which was recently introduced for PM implantation by our group (26), were evaluated. No significant correlations were found for any timepoint or parameter (Pearson’s r between 0.339 and −0.170, *p* > 0.05 for all correlations). Detailed results are summarized in Table 3, and scatter plots of these correlations are presented in Figure 5.

**Table 3:** Correlations between AS-OCT-derived structural and AS-OCTA-derived vascular bleb parameters.

Variables	Pearson r [95% CI]	*p*-value
1 W BWT / 1 W BVD	0.339 [−0.136, 0.688]	0.1553
1 W TBH / 1 W BVD	0.191 [−0.288, 0.594]	0.4330
2 W BWT / 2 W BVD	0.119 [−0.368, 0.555]	0.6383
2 W TBH / 2 W BVD	−0.170 [−0.590, 0.322]	0.4990
4 W BWT / 4 W BVD	−0.081 [−0.516, 0.387]	0.7409
4 W TBH / 4 W BVD	−0.077 [−0.514, 0.391]	0.7527

BWT, bleb wall thickness; BVD, bleb vessel density; TBH, total bleb height.

## Discussion

In this study, we analyzed the predictive value of two quantitative, AS-OCT-derived bleb parameters (BWT and TBH) in the early postoperative phase for secondary intervention after PM implantation. Neither BWT nor TBH predicted secondary interventions after PM implantation, and neither correlated with AS-OCTA-measured bleb vascularity (BVD) at 1, 2, or 4 weeks after primary surgery. The latter analysis was performed because we previously identified BVD at 2 and 4 weeks after primary surgery as an early predictive marker for future secondary intervention up to 1 year after PM implantation in the same cohort as in this study (2/4 weeks logistic regression *p*-values: 0.0244/0.0098, 2/4 weeks AUROCs: 0.796/0.909) (26).

In an effort to find objective markers for bleb function AS-OCT has become an increasingly popular tool both in clinical research and routine care (14, 29). Concerning bleb evaluation after PM implantation, three studies have evaluated correlations between quantitative bleb markers and IOP and obtained inconsistent results (23–25). In 2022, Ibarz Barbera et al. found no correlations between IOP and horizontal or vertical (TBH minus BWT) bleb dimensions in a longitudinal study with up to 3 months of follow-up (24). Later, the same group analyzed various bleb parameters in a cross-sectional design 1 year after PM implantation and reported a significant correlation between TBH and BWT with surgical success (defined by an IOP between 6 and 17 mmHg and a > =20% reduction without medication) in a univariate analysis, which did not persist in a multivariate analysis (25). Finally, in a retrospective cross-sectional analysis, Gambini et al. did not find associations between bleb dimensions and IOP 6 months after PM implantation (23).

The course of BWT and TBH in our cohort is, however, comparable to the published values by Ibarz Barbera et al. after PM implantation, showing a noticeable increase in both parameters during the first 3–6 months, transitioning to a plateau-like phase in the second half of the 1-year follow-up period (25).

The available body of evidence for predictive AS-OCT biomarkers after TE contrasts with our results for PM implantation. Narita et al. found TBH and BWT after 2 weeks to be predictive of surgical success (defined by an IOP < = 15 mmHg and a > 20% reduction without secondary intervention) up to 1 year after surgery (16). Waibel et al. detected higher BWT as early as 1 week and higher bleb cavity height (TBH minus BWT) 2 weeks after TE in functioning blebs compared with non-functioning blebs over a 3-month follow-up period (15). These findings are supported by further studies showing concomitant associations (at the same timepoints) between quantitative AS-OCT bleb parameters and IOP (or IOP-based success endpoints) after TE (17–22).

A possible reason for the discrepancy between TE and PM implantation could be differences in bleb position and morphology, as recently reported by Hasan et al. in an extensive comparative study of functioning blebs after TE and PM implantation (30). Compared to TE blebs, PM blebs showed fewer conjunctival microcysts, a more consistent Tenon’s capsule appearance, and a more posterior drainage (through the posterior PM tip, where Tenon’s capsule is thicker). Based on these findings, the authors suggested differences in bleb drainage between the two surgical techniques (30).

In this context it appears also notable that the vascular parameter BVD, which is independent of bleb thickness, was recently found to be an early predictive marker for secondary intervention after PM implantation by our research group (26). This result aligns with AS-OCTA-based vascularity biomarkers after TE (31–33), which could be indicative for a more procedure-independent validity of bleb vascularity biomarkers.

Studies correlating or combining structural AS-OCT-based and vascular AS-OCTA-based bleb parameters are rare and currently available for TE and XG implantation but not for PM implantation. Yin et al. found a correlation between vessel area and TBH 1 month after TE (33). Hayek et al. analyzed preoperative conjunctival vessel density and found an association with postoperative microcysts (32). Six months after XG implantation, Mastropasqua et al. detected a correlation between TBH and bleb wall microcysts with vessel displacement area, a marker for flow voids (34). In an effort to combine structural and angiographic data Luo et al. designed an objective bleb evaluation score in which information on vessel density, bleb height and microcysts were included (35).

To the best of our knowledge, the current study is the first to correlate AS-OCT-based structural and AS-OCTA-based vascular bleb parameters after PM implantation. While none of these correlations are statistically significant, this finding is not surprising, as BVD at two of the three presented timepoints (postoperative weeks 2 and 4) was recently shown to be an early predictor of secondary intervention after PM implantation in the same cohort (26), and BWT/TBH, as presented in this study, are not. The discrepancy in BWT/TBH data between TE and PM implantation, as discussed above, might also explain the diverging results between the study of Yin et al. and our analysis (33).

This study has several strengths and limitations. Strengths include the concomitant presentation and correlation of AS-OCT- and AS-OCTA-based parameters, the prospective study design, and the concise, clinically relevant endpoint definition. While the two earlier points have already been thoroughly discussed, the latter shall also be briefly outlined. We deliberately refrained from including IOP in our endpoint definition, which is commonly used in comparable studies. Instead, we relied solely on the clinically relevant necessity for secondary intervention, which reflects both IOP and clinical bleb appearance. This was done to avoid misclassification as bleb failure solely based on concepts such as threshold IOP or percent IOP reduction as both may be arbitrary and definitely depend on baseline IOP.

An obvious limitation of this study is its relatively small sample size, particularly given that we present non-significant, negative results and draw conclusions from them. As mentioned above, the sample size in the present study would, however, at least have allowed for the detection of an association if the minimum / maximum values for either parameter modified this risk by 20%, assuming a baseline secondary intervention incidence of 30%. Furthermore, we consider the type II error risk to be low due to the following two circumstances: (1) AS-OCT studies after TE with comparable sample sizes detected differences in bleb thickness parameters (15, 19) and (2) we recently found BVD 2 and 4 weeks after PM implantation to be a predictor for secondary intervention in the same cohort (26). As a consequence of the small sample size, a multivariate analysis of the data was not statistically feasible. We, however, acknowledge that various confounders (e.g., baseline IOP, glaucoma subtype, number of preoperative MEDs, age, and early postoperative IOP) might influence our results and should therefore be tested in future confirmatory studies with larger sample sizes.

Another limitation of the study is the complex relationship between clinical bleb appearance, AS-OCT(A)-based bleb parameters, and IOP. As discussed in this report and in recent reviews (14, 29), a plethora of information is available for cross-sectional study designs, but less so for longitudinal studies. The analysis of the study was focused on the predictive value of AS-OCT(A)-based bleb features at a very early postoperative timepoint for a clinical decision (needling/revision) made later in the postoperative course. Inherent to this analysis is a potential confounding effect of the clinical appearance and IOP.

Finally, due to the pilot-study character of this analysis, no formal evaluation of interobserver or intraobserver variability of AS-OCT- and AS-OCTA-based bleb parameters was performed.

In conclusion, structural AS-OCT-based bleb thickness parameters (BWT and TBH) in the early postoperative phase were not found to be associated with future secondary intervention after PM implantation and did not correlate with AS-OCTA-derived BVD. The discrepancy with the value of quantitative AS-OCT parameters after TE and with recently published AS-OCTA-derived vascular parameters after PM implantation may be related to differences in bleb drainage between TE and PM. The findings of this study, together with the recently published angiographic data in the same cohort, suggest that AS-OCTA-derived BVD could be a better predictor for secondary intervention after PM implantation than BWT/TBH. Larger studies are necessary to confirm these hypothesis-generating results. Also, the role of preoperative vascularization needs further study.

## Data Availability

The raw data supporting the conclusions of this article will be made available by the authors, without undue reservation.
